# Classification of gait phases based on a machine learning approach using muscle synergy

**DOI:** 10.3389/fnhum.2023.1201935

**Published:** 2023-05-17

**Authors:** Heesu Park, Sungmin Han, Joohwan Sung, Soree Hwang, Inchan Youn, Seung-Jong Kim

**Affiliations:** ^1^Biomedical Research Division, Korea Institute of Science and Technology, Seoul, Republic of Korea; ^2^Department of Biomedical Engineering, Korea University College of Medicine, Seoul, Republic of Korea; ^3^Division of Bio-Medical Science and Technology, KIST School, Korea University of Science and Technology, Seoul, Republic of Korea; ^4^School of Biomedical Engineering, Korea University, Seoul, Republic of Korea

**Keywords:** muscle synergy, neurological, muscle module, gait phase, electromyography (EMG)

## Abstract

The accurate detection of the gait phase is crucial for monitoring and diagnosing neurological and musculoskeletal disorders and for the precise control of lower limb assistive devices. In studying locomotion mode identification and rehabilitation of neurological disorders, the concept of modular organization, which involves the co-activation of muscle groups to generate various motor behaviors, has proven to be useful. This study aimed to investigate whether muscle synergy features could provide a more accurate and robust classification of gait events compared to traditional features such as time-domain and wavelet features. For this purpose, eight healthy individuals participated in this study, and wireless electromyography sensors were attached to four muscles in each lower extremity to measure electromyography (EMG) signals during walking. EMG signals were segmented and labeled as 2-class (stance and swing) and 3-class (weight acceptance, single limb support, and limb advancement) gait phases. Non-negative matrix factorization (NNMF) was used to identify specific muscle groups that contribute to gait and to provide an analysis of the functional organization of the movement system. Gait phases were classified using four different machine learning algorithms: decision tree (DT), k-nearest neighbors (KNN), support vector machine (SVM), and neural network (NN). The results showed that the muscle synergy features had a better classification accuracy than the other EMG features. This finding supported the hypothesis that muscle synergy enables accurate gait phase classification. Overall, the study presents a novel approach to gait analysis and highlights the potential of muscle synergy as a tool for gait phase detection.

## 1. Introduction

Human gait is a complex cyclical activity, involving a coordinated interaction between the central nervous system, muscles, and bones of the lower limb (Charalambous, 2014). In human locomotion analysis, the gait cycle is defined as the period between the initial contact of one foot and the subsequent occurrence of the same event with the same foot. Several partitioning models have been proposed based on distinct clinical objectives. Typically, gait partitioning models consist of two primary phases: swing and stance (Jasiewicz et al., 2006; Catalfamo et al., 2008; Formento et al., 2014); however, some models include three or more phases (Abaid et al., 2013; Agostini et al., 2014; Taborri et al., 2014, 2015). Accurate detection of the gait phase is essential for precise and accurate control of lower limb assistive devices, such as prostheses (Kumar et al., 2022), exoskeletons (Nazari et al., 2021), or other walking assistive devices (Nahavandi et al., 2022). Furthermore, these techniques are crucial in the domain of monitoring and diagnosing diverse neurological and musculoskeletal disorders, such as Parkinson’s disease, stroke, and cerebral palsy (Handelzalts et al., 2019).

A variety of wearable sensors, including accelerometers (Sant’Anna and Wickström, 2010; Rueterbories et al., 2014; Boutaayamou et al., 2015), gyroscopes (Mannini et al., 2014; Gouwanda and Gopalai, 2015; Taborri et al., 2015), and inertial measurement units (IMUs) (Kotiadis et al., 2010; Mariani et al., 2013; Hundza et al., 2014), have been used in previous studies to perform gait phase detection. However, wearable sensor-based kinematic analysis has limitations in analyzing the effects of muscle compensation during abnormal gait (Tan et al., 2020). In contrast, electromyography (EMG) signals have the potential advantage of being more closely related to the underlying neuronal control mechanisms involved in normal and pathological walking (Chvatal and Ting, 2013; Frère, 2017). However, although EMG signals offer basic information into muscle activation patterns, they do not directly reveal the modular organization of the neuromuscular system. Furthermore, the high dimensionality and complexity of EMG signals present challenges in interpreting the underlying motor control mechanisms. Thus, investigating the modular control of muscles is essential for a deeper understanding of the neuromuscular system’s role in gait.

Muscle activity during gait has been observed to cluster into sets of co-activated muscles, referred to as muscle synergies or modules (Clark et al., 2010). The muscle synergy approach has been widely used to monitor the alterations in the EMG characteristics of patients with gait disorders (Singh et al., 2018). Although the specific mechanism by which muscle synergies reflect the process of central nervous system control of distal motor function is still under debate, the consensus is that modularization of muscle activity can lower the computational complexity involved in selecting motor coordination strategies. Identifying muscle synergies during a gait reveals how the central nervous system coordinates the recruitment of different muscle groups (Hagio et al., 2015). In previous studies for healthy individuals, it was investigated whether muscle synergies vary depending on walking speed and walking environment, such as flat ground, underwater, and slope walking (Yokoyama et al., 2016; Saito et al., 2018a; Dewolf et al., 2020). From a rehabilitation perspective, some studies have observed that kinematic coordination is restored through changes in neuromuscular coordination in musculoskeletal patients (Routson et al., 2013; Ardestani et al., 2019).

Various methods can be implemented for detecting gait phase by training processed input signals. Lately, artificial neural networks (ANNs) (Choi et al., 2022) and deep learning (DL) (Nazari et al., 2022) techniques have seen a marked increase in popularity, driven by improvements in computational power and data accessibility. Prominent examples of these techniques include the convolutional neural network (CNN) (Shi et al., 2022), long short-term memory (LSTM) (Tran et al., 2021), and CNN-LSTM (Zhu et al., 2021). However, these models have many learnable parameters, requiring ample training data for accurate development. Moreover, these models need labeled data for learning, which demands data collection in diverse controlled environment, potentially limiting their applicability (Tran et al., 2021; Zhu et al., 2021; Shi et al., 2022). In this study, gait phase classification was performed using machine learning techniques, with feature values extracted from the EMG signals, addressing the limitations of the aforementioned models.

The purpose of the study was to investigate whether muscle synergy can be effectively used as a feature for gait event classification. We used a machine learning-based approach to validate the performance of the gait event classification. Specifically, this study used a support vector machine (SVM) algorithm to classify gait events and predict phase transition times. Two different classification schemes (3-class and 2-class) were also used to evaluate the gait phase classification. Finally, we compared the performance of the gait phase detection using different feature extraction methods across different gait events.

## 2. Materials and methods

### 2.1. Signal acquisition

Eight healthy individuals (mean ± standard deviation age, 59.25 ± 21.92 years; height, 169.25 ± 8.10 cm; body mass, 75.96 ± 16.90 kg; BMI, 26.34 ± 4.46 kg/m^2^) were recruited as subjects to collect gait data. Only volunteers with no history of neurological or musculoskeletal disorders and with a resting blood pressure within the range of 90/60 to 170/90 mm Hg were eligible to participate in this study. The experimental task was approved by the institutional review board at the Korea Institute of Science and Technology (IRB 2021−015).

**FIGURE 1:**
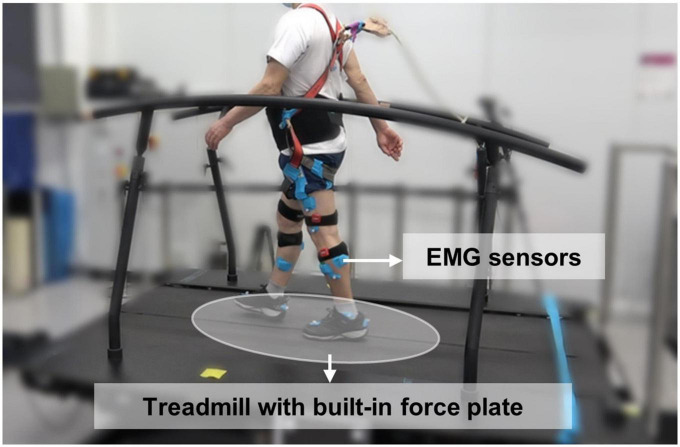
Gait experimental environment with attached EMG sensors and treadmill.

Wireless EMG sensors (Trigno Avanti, Delsys, Natick, MA, USA) were attached to measure the EMG signals observed in the lower extremities during walking. EMG sensors were attached to four muscles in each lower extremity, and these muscles were tibialis anterior (TA), soleus (SOL), gastrocnemius lateralis (GL), and rectus femoris (RF). Only the data from each individual’s dominant leg were used for further analysis. Subjects performed the gait on a treadmill with built-in force plates (M-gait, Motek, Amsterdam, Netherlands) to measure the gait cycles and events (Figure 1). To help the subjects adapt to walking on the treadmill, they experienced both a gradual increase in speed from a slower pace and a gradual decrease in speed from a faster pace. Then, the preferred speed, which the subjects considered to be the same as their usual walking speed, was measured, and on average, the gait speed was 0.84 ± 0.30 m/s. Both the EMG and force plate data were acquired at 2000 Hz during a 1 min walk at the preferred speed.

### 2.2. Data preparation

Raw EMG signals were band-pass filtered (zero-lag Butterworth band-pass filter, cut-off frequency 20−350 Hz) to remove high-frequency noise and motion artifacts. The filtered signal was then applied with full-wave rectification. Then, a fourth-order Butterworth low-pass filter was used with a cutoff frequency of 5 Hz. The ground reaction force (GRF) was measured from the force plates to detect the heel strike and toe-off time points, which were then used for the gait cycle analysis. The GRF signals were then processed to identify the gait phases. To determine whether the proposed method can accurately classify walking phases, both 3-class and 2-class dividing methods were adopted. The 3-class gait phases consist of weight acceptance (WA), single limb support (SS), and limb advancement (LA). A way to separate the gait phases into 2-class is to divide them into a stance phase (ST) and a swing phase (SW).

**FIGURE 2:**
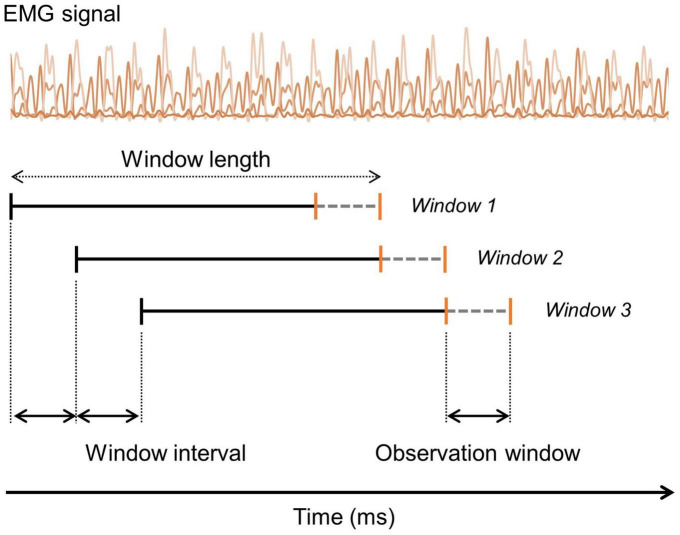
Illustration of the segmentation and labeling process of the EMG signals. Segmentation is performed using an overlapping sliding window method with a specified window interval size. Labeling is done based on the ground-truth value within the observation window (gray dashed line).

Electromyography signals were segmented in overlapping sliding windows, and the windows had an interval of 5 samples (window interval). Each window was labeled based on the last five sample (observation window) data. Figure 2 shows the segmentation method used. Predicting the current gait state based on past segments is a fundamental principle in real-time gait intent recognition and gait event detection related fields. Each EMG window was labeled as WA, SS, and LA according to the reference gait phases based on the GRF data in the observation window in the case of the 3-class gait phases. In the case of the 2-class gait phases, each window was labeled as ST and SW according to the reference data of the observation window. When labeling of the different phases was observed within one observation window, the most frequently used phase was used for the labeling. The GRF signals were downsampled from 2000 to 400 Hz, to make the GRF and predicted gait phase samples comparable and enable synchronization.

### 2.3. Feature extraction

For the segmented EMG data set five types of feature vectors were defined.

(1) Time domain features (TD).
(2) Wavelet transform coefficients (WC).
(3) Wavelet transform features (WF).
(4) Raw EMG features (RE).
(5) Muscle synergy features (MS).

The features of the time domain consisted of a mean absolute value, number of slope sign changes, number of zero crossings, waveform length, fourth-order autoregressive, and root mean square. Wavelet transform coefficients were obtained through discrete wavelet decomposition using the Mallat algorithm (Mallat, 1989). As for wavelet transform features, five types of features from the wavelet transform coefficients (energy, variance, standard deviation, waveform length, and entropy) were extracted. The wavelet and scaling functions of the discrete wavelet transform were symlet7, and the depth of the decomposition level was set to 5. For the RE features, each element was extracted as the signal value of 4 muscles in that single window. Thus, the first element of the vector is the EMG values for muscles 1 through 4 of that segments. The subsequent vectors are the EMG values of the four muscles calculated in the second segment, and so on.

The EMG signals of each window were used for the muscle synergy analysis. Before calculating the muscle synergy, min-max normalization was performed on the EMG signals of each window, thus mapping the values in the 0−1 intervals. Muscle synergies for each subject were extracted using Non-Negative Matrix Factorization (NNMF) (Lee and Seung, 1999). The reason for using NNMF was because the feature matrix contains non-negative values, and the NNMF enforces non-negativity constraints during extraction of the non-negative synergy matrix. The NNMF minimizes the residual between the initial matrix and its decomposition as follows:


E=W⁢H+e,

where *E* is the *p* × *n* matrix (in which *p* is the number of muscles and *n* is the number of time points); *W* is the *p* × *k* matrix of the synergy weights containing spatial components of muscle coactivations; *H* is the *k* × *n* matrix of the synergy activation coefficients including the temporal information of the synergy coactivation; *k* is the number of extracted muscle synergies, and *e* is the residual error. To avoid local minima, the NNMF algorithm was performed 20 times for each subject (Hug et al., 2011). Muscle synergy weights were normalized to the maximum value under the synergy they belong to. As for the MS, the synergy weights when the number of synergies was one were used. These feature extraction methods extracted multi-dimensional vectors, such as the TD with a dimensionality of 36, WC with a dimensionality of 1786, WF with a dimensionality of 120, RE with a dimensionality of 1600, and MS with a dimensionality of 4.

The synthetic minority oversampling technique (SMOTE) was adopted to address class imbalance in the dataset. SMOTE is a technique that creates new synthetic data points for the minority class to increase its representation in the dataset (Chawla et al., 2002). This technique generates oversampled data for the minority class by creating new instances in the feature space, using information from its K-nearest neighbors. This algorithm helps to overcome the overfitting problem posed by random oversampling. SMOTE was employed to balance the class distribution by increasing the number of samples in WA, SS, and SW datasets.

### 2.4. Optimal window length and classifier

To optimize the classifier, preliminary experiments were conducted to optimize the window length and to select the appropriate gait phase classifier. The accuracy of the four classification methods was compared: decision tree (DT), k-nearest neighbors (KNN), support vector machine (SVM), and neural network (NN). DT is a classification algorithm that presents patterns as predictable rules and provides visually readable results (Quinlan, 1986). KNN is a simple and efficient algorithm that classifies based on distance. It assigns a class to a datum based on the class of the k nearest data points. This method is useful when the properties of a data distribution are unknown (Al-Faiz et al., 2010). SVM is a statistical learning method that identifies hyperplanes to distinguish data belonging to two classes in an optimum way. SVM is easily applied to large and complex datasets, and it can be used for linear and non-linear data (Oskoei and Hu, 2008). NN is an efficient structure designed to mimic the decision-making ability of the central nervous system. Due to its ability to solve complex problems and patterns, it has been widely applied in many areas for classification (Ibrahimy et al., 2013). The size of the window used to segment the signal that is input to the classifiers can affect the classification or prediction performance (Nardo et al., 2020). For this reason, classification accuracy was compared with different window lengths of 100, 200, 400, and 600 ms. A fourfold evaluation was performed to compare the accuracy of the different classifiers and window lengths. The training set was composed of the signal windows from 6 subjects, and the remaining 2 subjects were used for testing. The hyperparameters of the model were optimized for each trial based on the training dataset and random search method.

**FIGURE 3:**
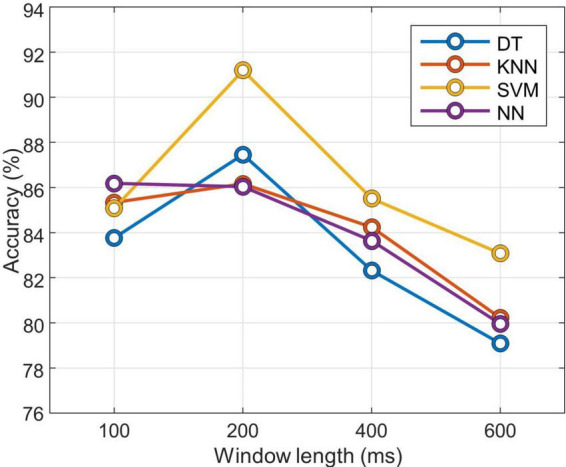
Classification accuracy according to window length for each classifier. DT, decision tree; KNN, k-nearest neighbors; SVM, support vector machine; NN, neural network.

After the training, it was evaluated on the test dataset, and the classification accuracy was recorded for different window lengths. Figure 3 shows the average classification accuracy by window length. It was confirmed that the result trained by the SVM with a window length of 200 ms had the best classification performance. All of the other classifiers except NN showed the highest accuracy when the window length was 200 ms. Therefore, the rest of the experiments were performed by setting the window length to 200 ms.

### 2.5. Gait phase classification

**FIGURE 4:**
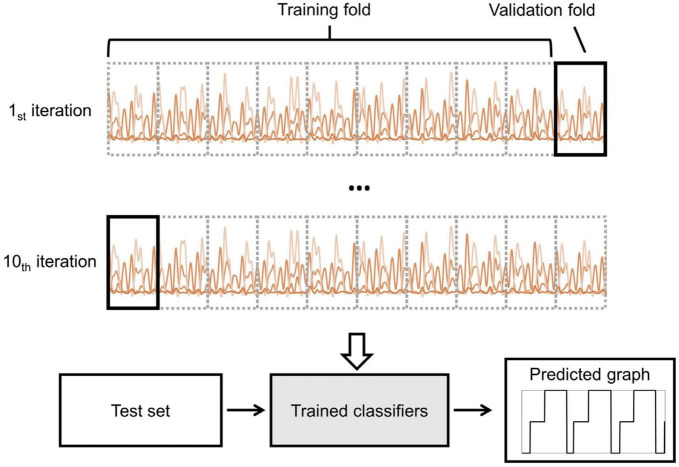
Illustration of the 10-fold cross-validation and prediction processes. The training dataset, consisting of 90% of the data, was divided into a training fold, while the remaining 10% was allocated to the validation fold. The intra-subject approach was performed using the training dataset. The trained classifier was then used to predict the gait stage using the test dataset.

To evaluate the validity of the gait phase classification performance, a 10-fold cross-validation strategy was performed. As shown in Figure 4, the features of each subject were divided into a training set (90%) and a test set (10%). The training set was then divided into two groups: a 90% training fold and a 10% validation fold. Subsequently, the data from each subject was divided into three groups: training, validation, and testing. There was no overlap of data between groups. To find the best number of training epochs, an early stopping technique was used. If there was no improvement in accuracy on the validation set for ten consecutive epochs, the training process would stop.

The accuracy of the gait phase classification was compared according to the feature extraction method used. Specifically, we compared the classification accuracy of each subject when classifying gait phases into 3 and 2 classes using feature vectors composed of TD, WC, WF, RE, and MS. The accuracy of classifying the gait phases using MS was compared for each classifier used in the analysis. After performing the phase detection for the different classifiers, the detection accuracy was evaluated for the 3-class classifications and 2-class classifications for each subject.

### 2.6. Gait event identification

Gait phase classification performance was based on its ability to detect changes in the gait phases. Thus, the predicted values were compared to the ground-truth values calculated using the GRF to assess the accuracy of detecting the phase transition time. A post-processing procedure was carried out to eliminate any misidentified predictions. This involved identifying and removing sections of the predicted signal that were significantly shorter than physiologically plausible. Previous research categorized the gait into three stages based on the occurrences of initial contact (IC), opposite final contact (OFC), and opposite initial contact (OIC) events, which were noted to correspond to 10%, 50%, and 100% of the mean gait cycle of healthy individuals, respectively (Neumann, 2016). From this perspective, transitions from WA to other gait events with a duration of less than 54.69 ms (≈5 % of the gait cycles) were excluded. In both the SS and LA sections, any transitions that had a duration of less than 218.75 ms (≈20 % of gait cycles) were excluded. Based on the gait transition period of the final contact (FC) and initial contact (IC) events, the gait is divided into two stages occurring at 60% and 100% of the gait cycle, respectively. In both the ST and SW sections, any transitions that had a duration of less than 218.75 ms (≈20 % of gait cycles) were also rejected (Nardo et al., 2020). After that, to verify the accuracy of detecting the gait phase transitions, the predicted values were compared with the ground-truth values obtained from the GRF. Three gait events (WA, SS, and LA) and two gait events (ST and SW) were considered. Predicted events falling within a tolerance range of ± 1 segment (± 2.5 ms) of the GRF-estimated events were considered successfully predicted. A tolerance window of 2.5 ms falls within the acceptable range of the estimation errors that are typically reported for commonly used gait event detection methods (Panebianco et al., 2018).

The difference in the gait phase transition times was calculated using the mean absolute error (MAE) for each feature extraction method. The classification performance was evaluated using the precision, recall, and F1-score. The precision is defined as follows:


$$P\,r\,e\,c\,i\,s\,i\,o\,n=\frac{T\,P}{T\,P+F\,P}$$

where *TP*s are the true positives, and *FP*s are the false positives. The recall is defined as follows:


$$R\,e\,c\,a\,l\,l=\frac{T\,P}{T\,P+F\,N}$$

where *FN*s are the false negatives. The F1-score is defined as follows:


$$F\,1-s\,c\,o\,r\,e=2\times \frac{P\,r\,e\,c\,i\,s\,i\,o\,n\times R\,e\,c\,a\,l\,l}{P\,r\,e\,c\,i\,s\,i\,o\,n+R\,e\,c\,a\,l\,l}$$

If the predicted gait event is the same as the ground-truth, it is recognized as a true positive. Otherwise, the predicted event is recognized as a false positive or false negative.

### 2.7. Statistics

In the statistical analysis, the differences among various classification and feature extraction methods were evaluated (Tables 1, 2). The statistical difference in data distributions, including MAE, precision, recall, and F1-score, was also assessed (Table 3). A one-way analysis of variance (ANOVA) with Tukey’s *post-hoc* test was employed when the data met the normality assumption. In contrast, for non-normally distributed samples, the Kruskal-Wallis test followed by the Dunn *post-hoc* test was utilized for comparative analysis. Values with *p* < 0.05 were considered significantly different.

**TABLE 1:** Prediction accuracy with respect to the subjects for the different classification methods.

A					B				
Subject	DT	KNN	SVM	NN	Subject	DT	KNN	SVM	NN
1	89.5%	88.9%	96.0%	92.5%	1	84.0%	83.5%	96.0%	89.3%
2	96.6%	96.5%	97.4%	97.2%	2	98.9%	98.4%	98.5%	99.2%
3	88.1%	83.2%	94.8%	85.9%	3	95.3%	93.4%	94.2%	94.5%
4	96.8%	91.6%	93.0%	86.6%	4	80.3%	79.2%	93.6%	83.1%
5	96.7%	94.8%	99.1%	87.6%	5	96.9%	100.0%	99.3%	99.9%
6	90.4%	82.3%	95.3%	87.2%	6	92.4%	91.0%	95.1%	92.2%
7	91.8%	96.2%	97.0%	94.8%	7	99.8%	99.8%	100.0%	99.8%
8	81.8%	87.8%	88.9%	88.6%	8	90.8%	86.7%	94.2%	90.9%
Avg.	91.5%	90.2%	95.2%	90.0%	Avg.	92.3%	91.5%	96.4%	93.6%
Std.	5.2%	5.6%	3.1%	4.2%	Std.	7.0%	7.9%	2.5%	6.0%

(A): 3-class gait phase classification. (B): 2-class gait phase classification. DT, decision tree; KNN, k-nearest neighbors; SVM, support vector machine; NN, neural network.

**TABLE 2:** Prediction accuracy for the different feature extraction methods using SVM.

A						B					
Subject	TD	WF	WC	RE	MS	Subject	TD	WF	WC	RE	MS
1	76.3%	76.2%	83.5%	70.4%	96.0%	1	78.8%	84.3%	83.5%	84.5%	96.0%
2	90.3%	85.5%	96.9%	96.6%	97.4%	2	95.7%	93.0%	96.6%	98.3%	98.5%
3	91.6%	87.5%	94.5%	96.1%	94.8%	3	93.0%	86.0%	96.8%	98.3%	94.2%
4	83.2%	87.5%	85.9%	88.2%	93.0%	4	79.8%	85.3%	85.7%	88.5%	93.6%
5	91.6%	92.5%	94.1%	90.6%	99.1%	5	94.6%	92.8%	95.2%	91.1%	99.3%
6	86.2%	85.1%	96.0%	95.6%	95.3%	6	91.1%	93.1%	98.2%	97.3%	95.1%
7	85.2%	86.7%	94.0%	90.3%	97.0%	7	95.8%	97.4%	95.0%	98.9%	100.0%
8	88.2%	89.3%	93.1%	93.8%	88.9%	8	98.0%	94.7%	96.7%	96.5%	94.2%
Avg.	86.6%*	86.3%*	92.3%	90.2%	95.2%	Avg.	90.9%*	90.8%*	93.4%	94.2%	96.4%
Std.	5.2%	4.7%	4.8%	8.6%	3.1%	Std.	7.4%	4.9%	5.6%	5.4%	2.5%

(A): 3-class gait phase classification. (B): 2-class gait phase classification. *Indicates a significant difference between all features, *p* < 0.05. TD, time domain features; WF, wavelet transform features; WC, wavelet transform coefficients; RE, raw EMG features; MS, muscle synergy features; avg., average; std., standard deviation.

**TABLE 3:** Mean absolute error (MAE), precision, recall, and F1-score with respect to gait events for different feature extraction methods.

A						
		TD	WF	WC	RE	MS
MAE (ms)	IC*	55.06 ± 13.86	29.69 ± 14.97	10.85 ± 6.15	19.56 ± 2.78	7.40 ± 2.75
	OFC*	40.20 ± 7.35	20.36 ± 7.94	18.48 ± 7.81	26.32 ± 8.76	12.03 ± 5.10
	OIC*	30.23 ± 18.76	27.42 ± 6.72	28.90 ± 6.60	29.78 ± 7.56	5.00 ± 1.86
Precision	IC*	0.71 ± 0.20	0.79 ± 0.15	0.91 ± 0.05	0.85 ± 0.14	0.90 ± 0.09
	OFC	0.93 ± 0.05	0.95 ± 0.03	0.95 ± 0.03	0.93 ± 0.07	0.96 ± 0.04
	OIC*	0.96 ± 0.03	0.97 ± 0.03	0.98 ± 0.01	0.97 ± 0.02	0.99 ± 0.02
Recall	IC*	0.81 ± 0.13	0.85 ± 0.08	0.89 ± 0.05	0.87 ± 0.11	0.98 ± 0.03
	OFC	0.92 ± 0.07	0.96 ± 0.03	0.97 ± 0.02	0.95 ± 0.04	0.94 ± 0.06
	OIC	0.94 ± 0.03	0.95 ± 0.03	0.98 ± 0.01	0.95 ± 0.04	0.97 ± 0.04
F1-score	IC*	0.74 ± 0.14	0.81 ± 0.09	0.90 ± 0.01	0.85 ± 0.08	0.94 ± 0.05
	OFC	0.92 ± 0.04	0.95 ± 0.02	0.96 ± 0.01	0.94 ± 0.05	0.95 ± 0.03
	OIC*	0.95 ± 0.02	0.96 ± 0.01	0.98 ± 0.01	0.96 ± 0.02	0.98 ± 0.03
**B**						
		**TD**	**WF**	**WC**	**RE**	**MS**
MAE (ms)	IC*	74.48 ± 25.08	22.25 ± 3.01	20.71 ± 9.94	24.10 ± 5.00	10.29 ± 2.33
	FC*	84.11 ± 28.15*	21.63 ± 7.10*	18.02 ± 5.07	37.94 ± 15.10	9.04 ± 4.13
Precision	IC	0.96 ± 0.03	0.97 ± 0.04	0.97 ± 0.03	0.97 ± 0.03	0.96 ± 0.06
	FC	0.89 ± 0.17	0.95 ± 0.03	0.94 ± 0.09	0.91 ± 0.11	0.94 ± 0.07
Recall	IC	0.95 ± 0.07	0.97 ± 0.02	0.97 ± 0.04	0.95 ± 0.05	0.95 ± 0.05
	FC	0.94 ± 0.03	0.95 ± 0.06	0.96 ± 0.04	0.96 ± 0.04	0.95 ± 0.08
F1-score	IC	0.95 ± 0.04	0.97 ± 0.02	0.97 ± 0.02	0.96 ± 0.04	0.96 ± 0.04
	FC	0.91 ± 0.11	0.95 ± 0.03	0.95 ± 0.05	0.93 ± 0.07	0.94 ± 0.06

(A): 3-class gait phase classification. (B): 2-class gait phase classification. *Indicates a significant difference between all features, *p* < 0.05. TD, time domain features; WF, wavelet transform features; WC, wavelet transform coefficients; RE, raw EMG features; MS, muscle synergy features; WA, weight acceptance; SS, single limb support, LA, limb advancement; ST, stance phase; SW, a swing phase.

## 3. Results

### 3.1. Gait phase classification

In the absence of oversampling, the classification models for the 3-class gait event classification were trained with an average of 2047, 9100, and 11602 samples of extracted features from WA, SS, and LA, respectively. The models were then tested with 227, 1011, and 1289 samples. For the 2-class gait event classification, the models were trained using 13877 and 8872 samples of extracted features from ST and SW, respectively, and tested with 1541 and 986 samples. However, with oversampling, the number of training samples from WA and SS was increased to 11602 on average. Subsequently, the classification models were trained using 11602 samples of extracted features from WA, SS, and LA for the 3-class gait event classification, and using 13877 samples of extracted features from ST and SW for the 2-class gait event classification. The testing was performed using the same number of samples as in the case without oversampling.

The detection accuracy was evaluated for the 3-class and 2-class classifications for each subject as shown in Tables 1, 2. Gait phase classification was performed using MS as feature vectors in all classifiers for comparison under the same conditions across the subjects. Both the 3-class and 2-class classifications showed the highest accuracy when using SVM (Table 1). On the other hand, NN (90.0%) and KNN (91.5%), which had the lowest classification accuracy, also showed over 90%, respectively. However, the difference in predictive accuracy among the classification methods was not statistically significant in the one-way ANOVA test for both 3-class and 2-class classifications [for 3-class, *F*_(3, 28)_ = 2.14, *p* = 0.12; for 2-class, *F*_(3, 28)_ = 0.95, *p* = 0.43]. The 3-class classification had an average performance of 91.72% for all the classifiers compared with the 2-class classification accuracy of 93.44%, which was 1.72% points higher.

Table 2 shows the detection accuracy using different feature extraction methods for each subject. The classifiers used to classify gait events were all SVM for the comparison under the same conditions for each subject. The average classification accuracy (± standard deviation) showed the highest performance when using MS for both the 3-class and 2-class gait event classifications. The differences in accuracy for 3-class and 2-class gait phase classification among feature extraction methods were found to be statistically significant in a one-way ANOVA test [for 3-class, *F*_(4, 35)_ = 3.69, *p* = 0.01; for 2-class, *F*_(4, 35)_ = 4.20, *p* = 0.03]. The *post-hoc* test results confirmed that the prediction accuracy of the MS method was significantly different from that of the WF method for both classifications (for 3-class, *p* = 0.02; for 2-class, *p* = 0.03). Additionally, for the 3-class classification, a significant difference between the MS and TD methods was observed (*p* = 0.03).

### 3.2. Gait event identification

**FIGURE 5:**
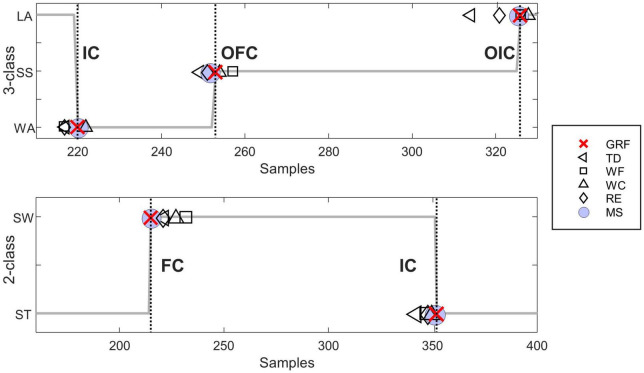
Representative figure comparing the gait event identification for each feature extraction method evaluated by a representative subject (subject seven). The top subplot represents the 3-class gait phase classification, and the bottom subplot represents the 2-class gait phase classification. The dotted line represents the moment each gait event occurred. Indicate the IC, initial contact; OFC, opposite final contact; OIC, opposite initial contact; FC, final contact; GRF, ground reaction force; TD, time domain features; WF, wavelet transform features; WC, wavelet transform coefficients; RE, raw EMG features; MS, muscle synergy features.

Figure 5 illustrates the distribution of accurately detected gait events compared to the events detected by GRF for each feature extraction method within each gait classification. Table 3 shows the difference between the predicted value and ground-truth of the phase transition time, calculated as the MAE in millisecond (ms). In addition, the statistical differences in the data distribution were represented by the precision, recall, and F1-score. The accuracy of the 3-class gait phase estimation for each feature extraction method was highest for the MS methods showing consistently high precision and recall values with average F-1 scores >0.94. The lowest average F-1 scores (0.94 ± 0.05) were observed for IC, whereas the highest average F-1 scores (0.98 ± 0.03) were observed for OIC, among the three gait events. The average MAE of the OIC was found to be the lowest (5.00 ± 1.86 ms) when using the MS features. The differences in MAE values for feature extraction methods were found to be statistically significant in one-way ANOVA and Kruskal-Wallis tests across all classes [for IC class, *F*_(4, 35)_ = 3.36, *p* = 0.02; for OFC class, *F*_(4, 35)_ = 0.68, *p* = 0.01; for OIC class, *H* = 10.77, *p* = 0.03]. The *post-hoc* test results revealed that the MAE of the MS method was significantly different from that of the TD method across all classes (for IC class, *p* = 0.02; for OFC class, *p* = 0.01; for OIC class, *p* = 0.03). Statistically significant differences in F1-scores for feature extraction methods were observed in the IC and OIC classes [for IC class, *F*_(4, 35)_ = 6.22, *p* = 0.02; for OIC class, *F*_(4, 35)_ = 2.92, *p* = 0.03]. The *post-hoc* test results consistently showed that the F1-score of the MS method was significantly different from the TD method (for IC class, *p* < 0.01; for OIC class, *p* = 0.04). Additionally, in the IC class, a significant difference was observed between the WF and MS methods (*p* < 0.05).

The mean and standard deviation of the performance scores for the 2-class gait phase detection are presented in Table 3. The F-1 score was found to be greater than 0.9 for all feature extraction methods used. The WF and WC feature extraction methods resulted in identical F-1 scores for the IC and FC, specifically 0.97 and 0.95, respectively. On the other hand, the MS features showed less MAE than the WC features, showing a higher agreement with the ground-truth. The F-1 scores for the IC and FC were found to be the lowest, at 0.95 or less, when using the TD feature extraction method. In addition, the MAE results indicated the highest error with an average value of 74 ms or more. The differences in MAE values for feature extraction methods were found to be statistically significant in the Kruskal-Wallis test across all classes [for IC class, *F*_(4, 35)_ = 3.36, *p* = 0.02; for FC class, *F*_(4, 35)_ = 0.68, *p* = 0.01]. The *post-hoc* test results confirmed that the MAE of the MS method was consistently different from both the TD and WF methods (for IC class, *p* = 0.02, 0.01; for OFC class, *p* = 0.01, <0.01; for OIC class, *p* = 0.04, 0.02). These results indicate that MS features can enhance estimation performance compared to TD or WF features when conducting gait phase detection.

## 4. Discussion

This study hypothesized that muscle synergy can provide more robust and informative features for gait event classification compared to traditional features such as the time-domain and wavelet features. To investigate this, a machine learning-based approach was used to classify gait events and predict phase transition times. The performance of four machine learning algorithms, DT, KNN, SVM, and NN, was compared in this study. The results showed that using MS features resulted in the highest classification accuracy for both 3-class and 2-class gait event classifications. Notably, the highest classification performance was observed when using a single muscle synergy, indicating the accurate representation of changes in gait patterns even in a low-dimensional MS. These experimental results support our hypothesis that muscle synergy, known as the coordinated activity of multiple muscles, enables accurate gait event classification. This study presents a novel approach to gait analysis, demonstrating the potential of muscle synergy as a tool for gait phase detection.

### 4.1. Gait cycle analysis and classification

It is widely acknowledged that the gait of humans involves three essential tasks, namely, WA, SS, and LA. These tasks must be executed successfully to achieve forward progression while maintaining balance (Perry and Davids, 1992). By dividing the gait cycle into two stages (ST and SW), changes in the activation patterns of the lower extremity muscles responsible for supporting body weight, stabilizing joints, propelling the body forward, and controlling the movement of the limbs can be observed during each cycle of gait. These tasks are performed during distinct and clearly defined phases of the gait cycle, which can be identified by ipsilateral and contralateral heel contact and toe-off events.

An intra-subject approach was used for the analysis in this study. The classifier was trained using 90% of the gait dataset, which was measured for 1 min, while the remaining 10% of the dataset was utilized as the test datasets. This process was repeated 10 times, and the test datasets were selected at different time segments (10-fold cross-validation). As a result, for the 3-class classification, the average accuracy was found to be 95.21 ± 3.14%, and the classification performance of all the subjects did not fall below 88.87% (subject 8). For the 2-class classification, the average accuracy was 96.37 ± 2.54%, and the lowest accuracy was high at 94.21% (subject 8). In other studies, related to muscle activity during the gait cycle, the intra-subject approach has also yielded encouraging results (Agostini et al., 2015; Castagneri et al., 2019). Because this model is not affected by inter-subject variations in muscle activation patterns and muscle strength, it is suitable for analyzing the effect of MS features on gait cycle prediction independently. On the other hand, because these models are trained on a specific individual, they may have limited generalizability to other individuals.

### 4.2. Muscle synergy analysis in gait

Muscle synergy can be conceptually viewed as a pattern of co-activation of muscle groups that can be combined to generate various motor behaviors. The concept of modular organization has proven useful in the study of locomotion mode identification (Hagio et al., 2015; Kibushi et al., 2018; Saito et al., 2018b; Esmaeili and Maleki, 2019) and rehabilitation of neurological disorders (Ting et al., 2019). Synergy weights are a fundamental component of muscle synergy analysis because they represent the underlying muscle patterns that are coordinated by the nervous system to produce a specific movement. These patterns related to gait were identified using NNMF. As demonstrated in Table 2, our findings exhibit a better classification performance compared with the raw EMG signals and commonly used features. These results indicate that the patterns of synergy weights during gait more accurately reflect the corresponding gait phases. The reason is that muscle synergy analysis enables the identification of specific muscle groups that contribute to gait and provides an analysis of the functional organization of the movement system. Furthermore, this approach enables the investigation of the underlying properties of the motor control system.

Several studies have suggested a relationship between the number of muscle synergies and walking performance. A reduced number of muscle synergies were observed during walking in the lower limb affected by chronic or subacute stroke compared to the unaffected limb (Clark et al., 2010). This finding suggests a decrease in the complexity of motor control, which is associated with poorer walking performance. Our results show that a low-dimensional muscle synergy features are sufficient to classify gait events. In this study, only one muscle synergy was extracted and used as the feature vectors for the comparative analysis. The features obtained by extracting two (with a dimensionality of 8) or three (with a dimensionality of 12) muscle synergies by performing NNMF showed the following results. The 3-class classification exhibited average performances of 95.21 ± 3.14%, 91.30 ± 5.63%, and 92.29 ± 4.53% when the number of synergies was 1, 2, and 3, respectively. In addition, the 2-class classification demonstrated an average performance of 96.37 ± 2.54%, 92.78 ± 6.12%, and 93.62 ± 4.67% when the number of synergies was 1, 2, and 3, respectively. Overall, the highest performance was observed when using a single muscle synergy. It has been confirmed that changes in gait patterns are accurately represented even in a lower-dimensional MS.

### 4.3. Limitation and future work

Furthermore, it should be noted that the experimental environment was limited as the subjects only performed the gait on a treadmill. In this study, a treadmill gait was utilized to collect the ground-truth data on the gait cycle by a built-in force plate and to measure the EMG signals during steady walking in a controlled environment. However, there are differences between treadmill walking and level ground walking in kinematics that can result from differences in foot placement, joint angles, muscle activation, and balance control (DeVita and Hortobagyi, 2000; Dingwell and Cavanagh, 2001; Riley et al., 2007; Sinclair et al., 2013). This study only including healthy adult males can also be considered as a limitation. Individuals with neuromuscular disorders or injuries may exhibit altered muscle activation patterns during their gait. Therefore, our model may not accurately predict the gait cycle for females or individuals with neuromuscular disorders or injuries. This study did not conduct a comprehensive real-time analysis, considering space, time, and computational complexities. This aspect may affect the effectiveness and efficiency of the proposed methods when applied in practical scenarios or assistive devices. Future studies should aim to obtain additional data by including a more diverse range of participants and testing them in various experimental environments. Additionally, the efficiency of computation for real-time analysis should be considered to enhance the applicability of the proposed methods.

Performing gait phase classification with EMG data has several clinical significances. Several studies have conducted gait phase classification using IMUs (Hundza et al., 2014; Tereso et al., 2014) or sensor fusion techniques (Lopez-Meyer et al., 2011; Novak et al., 2013; Rösevall et al., 2014); however, using EMG data offers the following advantages. It can provide insight into muscle activation patterns during gait, which can help identify muscle weaknesses or imbalances. It can also be used to monitor the progress of rehabilitation and track changes in muscle activation patterns over time. This information can be used to develop more effective rehabilitation programs and interventions for patients with gait disorders. The proposed technique of using muscle synergy to classify the gait cycle has many potential applications in different fields. For example, it can be used for recognizing gait intention in exoskeleton technology, remote diagnosis and digital therapy, and VR rehabilitation.

## Data availability statement

The raw data supporting the conclusions of this article will be made available by the authors, without undue reservation.

## Ethics statement

The studies involving human participants were reviewed and approved by the Institutional Review Board at the Korea Institute of Science and Technology (IRB 2021−015). The patients/participants provided their written informed consent to participate in this study.

## Author contributions

HP, SoH, JS, SuH, IY, and S-JK: conception and design of the experiments, drafting the article or revising it critically for important intellectual content, and final approval of the version to be published. HP and SuH: collection, analysis and interpretation of data. All authors contributed to the article and approved the submitted version.
